# Veterinary Surgery

**Published:** 1837-07

**Authors:** 


### VETERINARY SURGERY.

*Experimental Researches regarding Hydrophobia.* By M. Capello, of Rome.

The following observations are reported in a French journal by M. Furnari, but are the results of the labours of M. Capello.

M. Capello has published two memoirs, in which he endeavours to prove that hydrophobia, after its first transmission to another animal, does not preserve its poisonous properties. He brings forward a number of facts in support of this opinion. The following are a few of them.

1st. A dog spontaneously mad, bit a young man and an ox. The young man died five months after the bite with all the symptoms of hydrophobia. The ox three days after receiving the bite exhibited hydrophobic symptoms and was shot, but before this could be effected, he bit several animals of his own and of different species. Not one of these was affected with hydrophobia.

2d. A dog and cat were inoculated with the saliva of a dog affected with spontaneous hydrophobia. On the fourteenth day after inoculation the dog died, and on the thirty-fourth the cat died. With the saliva of these latter animals, another cat and dog were inoculated, but after suffering confinement for seven months, they were liberated, not exhibiting any hydrophobic symptoms.

3d. In the month of March, at Tivoli, a dog spontaneously mad, bit two other dogs; one was killed; the other attacked with symptoms of hydrophobia bit three or four women, but not one of them were ultimately injured.

4th. In January 1818, a dog belonging to M. Capuccini, was bitten by one labouring under hydrophobia. Thirty-eight days after, it exhibited premonitory symptoms and ultimately became mad, rushed out into the streets and bit four dogs and two children. It was proved that the dog first affected laboured under spontaneous hydrophobia. M. C. assured the owners of the four dogs, and the parents of the two children, that the dog who bit them laboured under hydrophobia the result of communication, or in the second degree, and that consequently they would be exempt. This prognostication was completely verified.

5th. Another case is related of a dog affected with this disease from communication and which bit many dogs and five individuals, but none of these ever presented symptoms of hydrophobia.

With regard to the cause of hydrophobia, M. Capello says it is neither rage, nor hot or putrid food, nor privation of liberty, nor excessive fatigue, nor suppressed perspiration; but only the venereal desire carried to excess and not satisfied. This causes great suffering, and he thinks this is quite sufficient to give rise to such a change in the economy of the animal as to produce hydrophobia. He is of opinion also, that the particular structure of the generative organs confirm this. In these animals the vesiculæ seminales are wanting, and consequently the prolific fluid cannot be excreted without copulation.

[If there is any truth in M. Capello's theory, respecting the cause of hydrophobia, it would be expedient, as a general rule, to have dogs castrated, leaving only a sufficient number to keep up the breed. The dogs used in dog-carts, for instance, would, we apprehend, draw equally well without their testicles, as horses do.] *Journal des Connaissances Medicales. November,* 1836.

